# Subtoxic Doses of Cadmium Modulate Inflammatory Properties of Murine RAW 264.7 Macrophages

**DOI:** 10.1155/2015/295303

**Published:** 2015-08-03

**Authors:** Sina Riemschneider, Martin Herzberg, Jörg Lehmann

**Affiliations:** ^1^Fraunhofer Institute for Cell Therapy and Immunology (IZI), 04103 Leipzig, Germany; ^2^Martin Luther University of Halle-Wittenberg, Institute for Biology/Microbiology, 06120 Halle, Germany

## Abstract

Cadmium (Cd) is a toxic heavy metal that exhibits various adverse effects in the human and animal organism. Its resemblance to essential metals such as calcium, iron, and zinc leads to an unintended uptake in cells after intake through inhalation and ingestion. In this study we investigated the toxicity and the immunomodulatory potential of Cd in nonactivated and activated murine macrophages (i.e., cell line RAW 264.7). Cadmium alone caused a dose-dependent decreased viability of exposed cells. Subtoxic Cd concentrations delayed cell death in macrophages, resulting from cytotoxic storm, producing reactive oxygen species (ROS) and nitric oxide (NO), in response to their stimulation by bacterial antigens via pattern-recognition receptors (PRRs). In addition, production of selected pro- and anti-inflammatory cytokines, the chemokine CXCL1 (KC), and NO was determined. We observed that proinflammatory IL-1*β* and also CXCL1 were highly upregulated whereas anti-inflammatory or regulatory cytokines IL-6 and IL-10 were suppressed by 10 *µ*M Cd. Also production of antibacterial NO was significantly reduced through exposure to 10 *µ*M Cd, maybe explaining better survival of macrophages. Additionally, we could show by analysis via ICP-MS that different effects of Cd in nonactivated and activated macrophages definitely did not result from different Cd uptake rates.

## 1. Introduction

Global industrialization has caused a dramatic contamination of the environment with toxic heavy metals such as cadmium (Cd), lead, or mercury representing a latent danger for men's health via the nutrition chain through contaminated water or food products [1, 2] or maternal milk [3]. Alarmingly, modern production methods in agriculture contribute more and more to Cd contamination of food products through phosphate fertilizers or mobilisation of biosolid-borne Cd by chloride ligands in soil solution [4], indiscriminate use of pesticides such as glyphosate [5], and the agricultural use of anaerobically digested residues from full-scale biogas plants [6]. Other crucial sources for accumulation of heavy metals, in particular cadmium, in men is cigarette smoking [2] and exposure of workers in industries [7]. Since Cd is capable of entering cells via Ca^2+^ channel [8], ZIP-transporters [9, 10] and divalent metal transporters 1 (DMT-1, Nramp2) [11, 12] of the cell membrane of many cells and accumulate intracellularly due to its binding to cytoplasmic and nuclear material constituting a potential threat to human and animal health [13, 14].

Although tremendous work has been done to identify diverse toxicological pathways of heavy metals, such as mutagenic, cancerogenic, teratogenic, reprotoxic, nephrotoxic, or neurotoxic effects [15–22], there is still fragmentary understanding how certain heavy metals affect the innate or adaptive immune system. However, those adverse effects may potentially impact human health in terms of suppressing immunity to infection and control of cancer or supporting the development of autoimmunity or allergy even at very low subtoxic exposure doses (reviewed in [23]).

Data from an* in vivo* infection model (i.e.,* Salmonella* Enteritidis) under Cd exposure showed a significant immunosuppressive effect of this heavy metal on the early and late immune response against infectious agents, suggesting that Cd influences both innate as well as adaptive immune mechanisms (Hemdan and Lehmann, unpublished). Interestingly, the phenotype of this mouse model was associated with a hyperactivation rather than a suppressed immune response* in vivo*. A first* in vitro* study to investigate the underlying mechanism using human peripheral blood mononuclear cells (PBMCs) has shown that the immunomodulating capacity of Cd depends significantly on the activation stimulus and the target cell population. Polyclonal activation of T cells and antigen-presenting cells (APCs) by anti-CD3/anti-CD28 or anti-CD40, respectively, versus activation of APCs via pattern-recognition receptor (PRR) ligands by heat-killed salmonellae (hk* S*.E.) resulted in completely different immunomodulatory effects [24]. However, the mode of action is still elusive. For the complete understanding of the immunomodulation by Cd a deeper knowledge of Cd-mediated cellular and molecular effects on individual cell types orchestrating the innate and adaptive immune response is essentially required. In the present study we attempted to characterize the influence of cadmium on the activity of murine macrophages* in vitro* to get an impression how activation of macrophages by multiple PRR ligation, induced by hk* S*.E., is modulated under cadmium exposure.

## 2. Materials and Methods

### 2.1. Cell Culture of Murine Macrophage Cell Line RAW 264.7

RAW 264.7 (ATCC TIB-71) represents a murine (BALB/c; H2^d^) adherent growing monocyte/macrophage cell line which is transformed by Abelson murine leukemia virus [25]. RAW 264.7 cells are capable of pinocytosis and phagocytosis, antibody-dependent lysis of tumor cells as well as nitric oxide (NO) and cytokine production and they are responsive to LPS [25]. Cells were cultured in 175 cm^2^ cell culture flasks (Greiner Bio-One, Frickenhausen, Germany) in phenol-red free RPMI 1640 medium supplemented with 10% FBS, 2 mM L-glutamine, 10 mM HEPES buffer, 100 *μ*g/mL penicillin/streptomycin (Biochrom, Berlin, Germany), and 50 *μ*M *β*-mercaptoethanol (Sigma Aldrich, Steinheim, Germany) at 37°C, 5% CO_2_, and 95% air humidity.

For experiments the cells were activated with heat-killed* Salmonella enterica* Serovar Enteritidis (SalmoVac SE, IDT Biologika GmbH, Dessau-Rosslau, Germany). The relative antigen concentration used in this study (ratio: 10^8^ hk* S.*E. to 10^7^ macrophages) was previously determined (data not shown) and ensures appropriate activation of macrophages.

### 2.2. Determination of Cadmium Content by Means of Inductively Coupled Plasma: Mass Spectrometry Analysis (ICP-MS)

Cells were plated at a density of 1 × 10^6^ cells/mL in 58 cm^2^ cell culture dishes with 10 mL of cell culture medium described above. Afterwards cells were incubated with 0.01 *μ*M, 0.1 *μ*M CdCl_2_, or 10 *μ*M CdCl_2_ (Sigma Aldrich) alone, or in the presence of 1 × 10^8^ hk* S.*E. After 2 h cells were harvested and washed twice with phosphate-buffered saline (0.15 M NaCl, pH 7.4; PBS) containing 5 mM EDTA (Sigma Aldrich). The supernatant was discarded and the residual liquid carefully removed at each step. The pellet was suspended in concentrated 67% (w/v) HNO_3_ (trace metal grade; BDH Prolabo, VWR, Darmstadt, Germany) and mineralized at 70°C for 2 h. Samples were diluted to a final concentration of 2% (w/v) nitric acid. Indium was added as internal standard at a final concentration of 10 ppb. Elemental analysis was performed via inductively ICP-MS using ESI-sampler SC-2 (Elemental Scientific Inc., Omaha, USA) and an X-Series II ICP-MS instrument (Thermo Fisher Scientific, Bremen, Germany) operating with a collision/reaction cell and flow rates of 5 ml/min of He/H_2_ (93%/7%), with an Ar carrier flow rate of 0.76 l/min and an Ar make-up flow rate of 15 l/min. An external calibration curve was recorded with ICP-multi element standard solution XVI (Merck, Darmstadt, Germany) in 2% nitric acid. The sample was introduced via a peristaltic pump and analyzed for its metal content. For blank measurement and quality/quantity thresholds, calculations based on DIN32645 TMM were used. The results were transformed from ppm, ppb, or ppt via molar units into atoms per sample and divided by the number of cells per sample.

### 2.3. Cytokine and Chemokine Detection by Enzyme-Linked Immunosorbent Assay (ELISA)

Cells were plated at a density of 1 × 10^6^ cells/mL in 58 cm^2^ cell culture dishes with 10 mL of cell culture medium described above. Afterwards cells were incubated with 0.1 *μ*M CdCl_2_ or 10 *μ*M CdCl_2_ and stimulated with 1 × 10^8^ hk* S.*E. Supernatants were collected and analyzed for their content of cytokines by ELISA. For determination of murine cytokines IL-1*β*, IL-6, IL-10, and TNF-*α* reagent sets and protocols of eBioscience (Frankfurt, Germany) were used. Concentration of the murine chemokine CXCL1 was measured using the reagent set and related protocol from R&D Systems (Wiesbaden, Germany). For determination of IL-6 and TNF-*α* supernatants were diluted 1 : 100 and 1 : 500, respectively. Finally, optical density signals were quantified using a conventional microplate reader and the concentrations were calculated in pg/mL by applying the Magellan Software 5 (Tecan Safire2, Tecan, Männedorf, Switzerland).

### 2.4. Measurement of Nitric Oxide (NO)

Cells were plated at a density of 1 × 10^6^ cells/mL in 58 cm^2^ cell culture dishes with 10 ml of cell culture medium described above. Afterwards cells were incubated with 0.1 *μ*M CdCl_2_ or 10 *μ*M CdCl_2_ and stimulated with 1 × 10^8^ hk* S.*E. Supernatants were collected and analysed for their content of nitrite and nitrate as stable final products of NO synthesis using the Griess reaction as described elsewhere [26]. Briefly, 50 *μ*L cell-free supernatants were mixed with 100 *μ*L of Griess reagent (1% sulfanilamide in ethanol absolute, 0.1% N-(1-naphthyl)-ethylenediaminedihydrochloride in 5% phosphoric acid, Sigma Aldrich). A calibration curve was prepared by means of serial dilution of sodium nitrite as calibration standard. The plate was incubated for 10 min at room temperature. Finally, concentration of nitrite/nitrate was quantified using a conventional microplate reader (Tecan Safire2, Tecan).

### 2.5. Real-Time Monitoring of Macrophage Adherence

Adherence of RAW 264.7 macrophages was monitored using the impedance-based xCELLigence RTCA system (xCELLigence RTCA SP instrument, ACEA, San Diego, CA, USA/Roche Diagnostics, Mannheim, Germany). For this method, a special 96-well electronic microtiter plate (E-Plate 96, ACEA/Roche Diagnostics) was used. The dimensionless cell index (CI) as an equivalent of the impedance measured in gold electrodes on the bottom of each well of the E-Plate 96 was continuously recorded over periods up to 200 h. For the measurement of impedance background, 50 *μ*L of cell culture medium was added to each well. After that 1 × 10^5^ cells were added to each well followed by addition of CdCl_2_ or additional hk* S*.E. to a final volume of 100 *μ*L/well. The E-Plate 96 were incubated at 37°C with 5% CO_2_ and monitored on the RTCA system. CI values were recorded every 30 min.

### 2.6. Determination of Cell Viability by WST-1 Assay

WST-1 assay (Roche Diagnostics) represents an easy-to-use method for evaluation of cell viability, proliferation, and cytotoxicity. The assay was performed in a 96-well microculture plate (Greiner Bio-One) according to manufacturer's instructions. Briefly, 1 × 10^5^ RAW 264.7 cells were added to each well followed by addition of CdCl_2_ or additional hk* S*.E. to a final volume of 100 *μ*L/well. After 24 h and 48 h 10 *μ*L of the water soluble tetrazolium salt WST-1 (2-[4-iodophenyl]-3-[4-nitrophenyl]-5-[2,4-disulfophenyl]-2H-tetrazolium, monosodium salt) was added in each well. In case of viable cells the tetrazolium salt is reduced to formazan and leads, therefore, to a change of colour [27]. Following incubation of the cells with WST-1 reagent the absorbance of supernatants was measured at 437 nm using a conventional microplate reader (Tecan Safire2, Tecan).

### 2.7. Statistical Analysis

SigmaPlot software (Systat, Erkrath, Germany) was used for statistical evaluation of results. Data were analyzed by one-way analysis of variance (ANOVA) and the Holm-Sidak's test was applied* post hoc*. Values were considered significantly different if *p* < 0.05.

## 3. Results

### 3.1. Dose-Dependent Effects of Cadmium on Cell Viability of RAW 264.7 Macrophages

**Figure 1:**
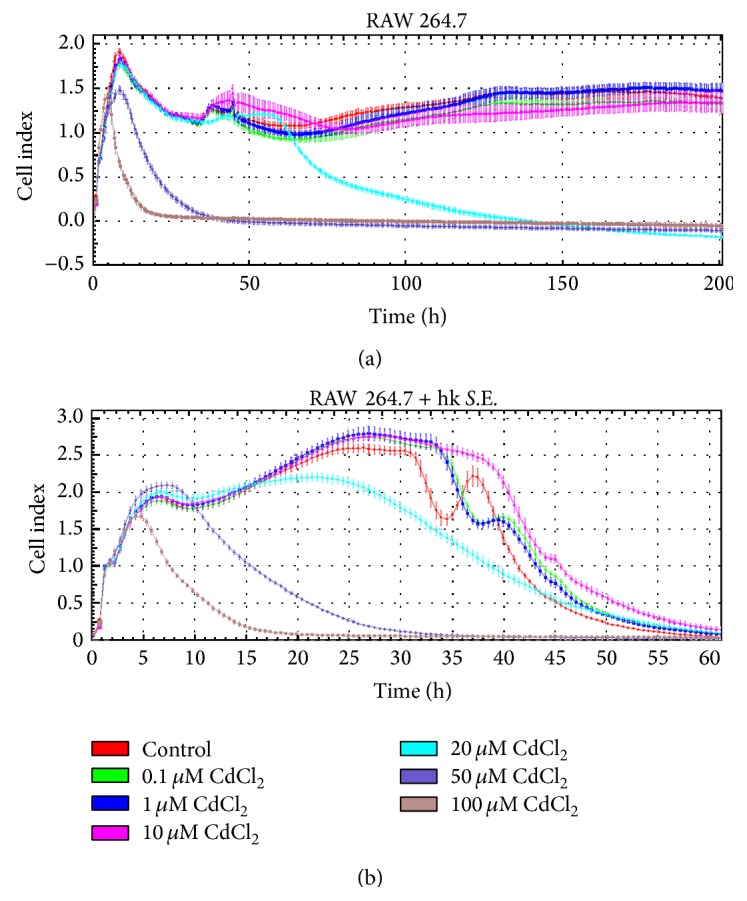
Influence of Cd on the adherence behavior of RAW 264.7 macrophages. Adherence and proliferation of RAW 264.7 cells were continuously monitored over a period of 200 h (a) and 60 h (b) using the xCELLigence RTCA system (ACEA). 2 × 10^5^ cells/well were seeded in a special 96-well plate (E-Plate 96) and subsequently stimulated with various Cd concentrations alone or in presence of hk* S*.E. Data represent the mean ± standard deviation (SD) and are representative of three independent experiments (*n* = 5-6 per experiment).

**Figure 2:**
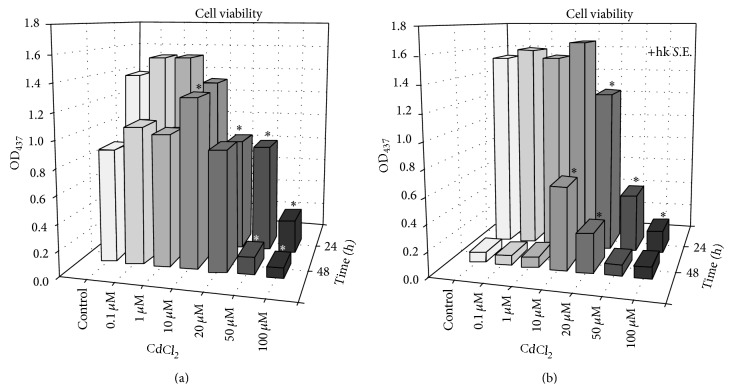
Influence of Cd on the cell viability of RAW 264.7 macrophages. Cell viability was determined using the WST-1 assay (Roche Diagnostics). 2 × 10^5^ cells/well were seeded in a 96-well microculture plate and subsequently stimulated with various Cd concentrations alone or in the presence of hk* S*.E. After 24 and 48 h WST-1 Reagent was added for 1 h followed by determination of the optical density in supernatants at 437 nm using a conventional microplate reader (Saphire2, Tecan). Data represent the mean values and are representative of three independent experiments (*n* = 2 per experiment). *∗* indicates significant (*p* < 0.05) differences compared to control.

In order to determine the subtoxic dose range of Cd, various Cd concentrations were studied using the impedance-based xCELLigence RTCA system (Figure 1(a)). The CI values show that incubation of RAW 264.7 macrophages with 100 *μ*M Cd caused rapid cell death as early as 5 h of exposure, while 50 *μ*M Cd impaired adherence of RAW 264.7 macrophages after 10 h and 20 *μ*M Cd reduced cell viability after 60 h. However, concentrations between 0.1 *μ*M and 10 *μ*M Cd did not cause changes of the adherence behaviour compared to untreated controls up to 200 h. A simultaneous stimulation of macrophages with hk* S.*E., a potent trigger of several PRRs, delivered a very similar result for 100 *μ*M and 50 *μ*M Cd (Figure 1(b)). A complete loss of adherence became apparent after 25 h of exposure with 20 *μ*M Cd. The cell viability of Cd-free control started to decrease after 32 h, whereas cell viability in cultures with 0.1 *μ*M and 1 *μ*M Cd showed a moderately prolonged survival since the decrease of CI signal started to decrease 2 h later compared to control. Additionally, 10 *μ*M Cd delayed the loss of adherence by 6 h compared to the control. In parallel, cell viability was also determined using the WST-1 endpoint assay. The results show a decreased cell viability of macrophages following exposure to 100 *μ*M and 50 *μ*M Cd for 24 h and 48 h in nonactivated and activated RAW 264.7 macrophages (Figures 2(a) and 2(b)). Additional stimulation of macrophages with hk* S*.E. led to WST-1 reduction only after 48 h exposure with 10 *μ*M Cd (Figure 2(b)).

### 3.2. Cadmium Uptake of Macrophages

To get an impression of the basal cadmium content in RAW 264.7 cells in culture and the amount of Cd uptake and accumulation after incubation of the cells in the presence of CdCl_2_ over a period of up to 20 h we determined the content of Cd as atoms per cell by means of ICP-MS. The result demonstrated a significant and continuous uptake and accumulation of Cd over time in the RAW 264.7 macrophages in dependence of the CdCl_2_ concentration in the culture medium (Table 1). The results in the presence or absence of hk* S*.E. were found to be very similar.

### 3.3. Effects of Cadmium on Cytokine, Chemokine, and NO Production

**Figure 3:**
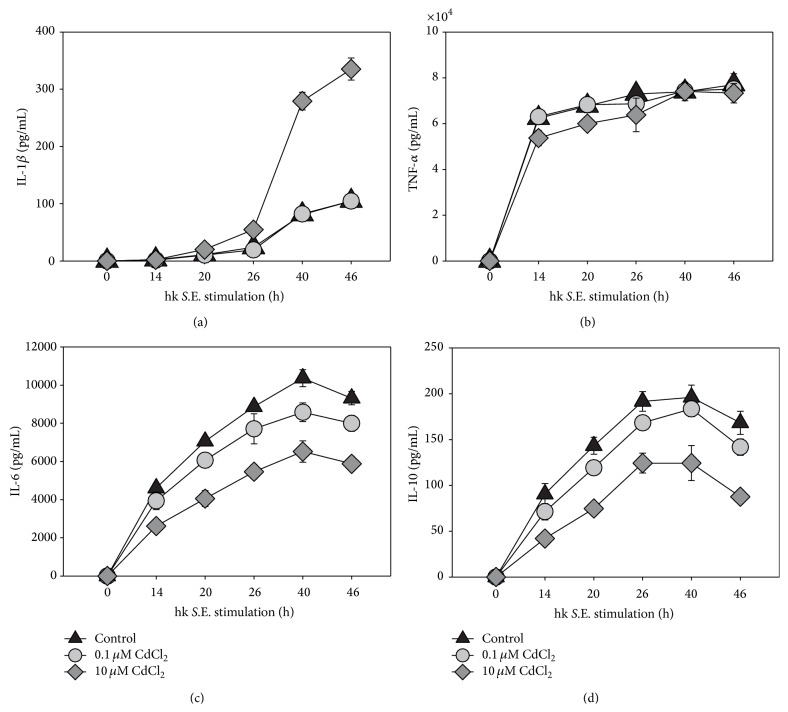
Influence of Cd on the cytokine production of RAW 264.7 macrophages. A volume of 10 mL containing 1 × 10^6^ cells/mL was seeded in a culture dish and subsequently stimulated with 0.1 *μ*M or 10 *μ*M Cd in the presence of hk* S*.E. After indicated periods of incubation supernatants were collected and cytokine (i.e., IL-1*β* (a), TNF-*α* (b), IL-6 (c), and IL-10 (d)) concentration was determined by ELISA. Data represent the mean ± S.E.M. and are representative of three independent experiments (*n* = 3).

**Figure 4:**
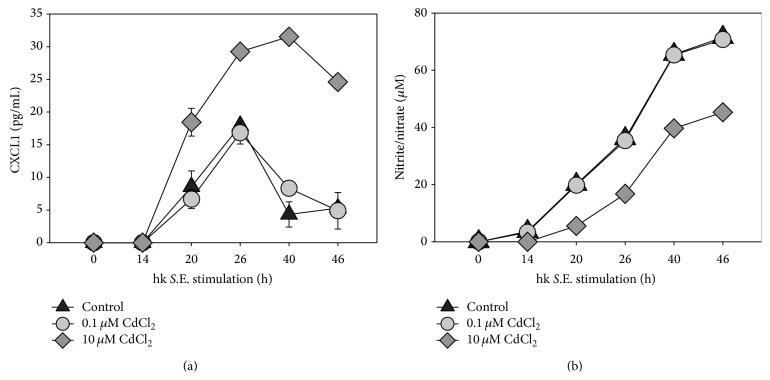
Influence of Cd on chemokine and NO production of RAW 264.7 macrophages. A volume of 10 mL containing 1 × 10^6^ cells/mL was seeded in a culture dish and subsequently stimulated with 0.1 *μ*M or 10 *μ*M Cd in the presence of hk* S*.E. After indicated periods of incubation supernatants were collected and chemokine (i.e., CXCL1 (a)) and nitrite/nitrate concentrations (b) were determined by ELISA or Griess reaction, respectively. Data represent the mean ± S.E.M. and are representative of three independent experiments (*n* = 3).

In order to identify immunoregulatory effects of Cd on macrophages the secretion of proinflammatory (i.e., IL-1*β* and TNF-*α*) and anti-inflammatory cytokines (IL-10 and IL-6) in response to Cd exposure with or without hk* S*.E. stimulation were measured by ELISA. Thereby it was of special interest to obtain data that may explain better survival of antigen-stimulated cells in the presence of 10 *μ*M Cd. Therefore, we compared cytokine concentrations in supernatants of cells exposed to 0.1 *μ*M or 10 *μ*M Cd in comparison to untreated controls (Figure 3). The results revealed that in case of IL-1*β* 0.1 *μ*M Cd exhibited no effect compared to control, whereas 10 *μ*M Cd led to a threefold increase of IL-1*β* concentration (Figure 3(a)). In terms of IL-10 and IL-6 0.1 *μ*M Cd led to a slightly decreased cytokine secretion, while 10 *μ*M Cd reduced the concentration of both cytokines by half (Figures 3(b) and 3(c)). The TNF-*α* secretion highly increased after hk* S*.E. stimulation, whereby the Cd exposition did not result in significant alterations of the cytokine production (Figure 3(d)). The secretion of chemokine CXCL1 was not effected by 0.1 *μ*M Cd compared to control, whereas 10 *μ*M Cd caused twofold increase of CXCL1 concentration in RAW 264.7 culture supernatants (Figure 4(a)). In contrast, NO production was found to be reduced by approximately 40% following exposure to 10 *μ*M Cd; however, 0.1 *μ*M Cd did not show any effect on this functional parameter (Figure 4(b)).

## 4. Discussion

Proinflammatory effects of Cd in subtoxic dose ranges have been shown in diverse human and murine cell lines or primary cells (reviewed in [28]). The upregulation of many cytokines such as IL-1*β*, IL-6, IL-8, and TNF-*α* illustrates an immunomodulatory potential of Cd [29, 30]. Therefore, the aim of this study was to determine the effects of Cd in a relevant* in vitro* model of bacteria-driven ongoing innate immune response. As macrophages play a key role in immunity to bacterial infections as bactericidal effector cells as well as APCs we started this complex of investigation with the study of macrophages. For reasons of standardization and comparability of immunotoxicological results we preferred to use the well-described and broadly accepted macrophage cell line RAW 264.7 as a highly standardized* in vitro* model rather than primary macrophages.

A basic requirement of an immunotoxicological* in vitro* model is the exact knowledge of the toxic and subtoxic dose ranges of the compound to be tested. This requires appropriate endpoints represented by classical cytotoxicity assays, such as MTT, XTT, WST-1, EZ4U, or LDH assay, in most cases. However, we have standardized a novel impedance-based real-time cell analysis method (i.e., xCELLigence RTCA and ACEA/Roche) for this purpose under GLP conditions. This methodology allows a very sensitive real-time monitoring of toxic effects mediated by Cd and other xenobiotic compounds with high time resolution and offers the opportunity of medium- and high-throughput testing. Thus, in addition to the endpoint WST-1 assay the viability of nonactivated and activated macrophages exposed to different Cd concentrations was determined using this method. Using endpoint assays, dose-dependent toxicity of Cd on organisms and cells have been shown by several groups [24, 31, 32]. These data could be underlined and significantly completed in terms of the time course of toxic Cd effects applying the impedance-based RTCA method in the present study. Here, concentrations about 10 *μ*M Cd caused reduced adherence and cell viability in nonactivated and activated macrophages compared to respective controls. However, when the cells were simultaneously exposed to hk* S*.E. as a bacterial antigenic stimulus, activating the macrophage via several PRRs (i.e., Toll-like receptor (TLR)2, TLR4, TLR7, and TLR9 [33]) 10 *μ*M Cd prevented the loss of adherence in comparison to control by 6 h as determined by xCELLigence RTCA. Also the WST-1 assay revealed viability of stimulated macrophages exposed to 10 *μ*M Cd after 48 h but not of control cells and cells exposed to Cd concentrations below 10 *μ*M. These results demonstrate that 10 *μ*M Cd is capable of delaying cell death caused by too strong activation through a potent bacterial antigenic stimulus, resulting in high production rates of cytotoxic proinflammatory cytokines, reactive oxygen species (ROS), and nitric oxide (NO).

In order to exclude that different effects in nonactivated and activated macrophages might be the result of different Cd-uptake rates, we determined Cd content in RAW 264.7 cells. Using ICP-MS analysis we could show that Cd content in RAW 264.7 macrophages is independent of cell activation with hk* S*.E. Furthermore, we observed an increase of Cd concentration per cell by exposure of increasing Cd concentrations in the cell culture medium as expected.

Previous reports on nonactivated human cells (i.e., human PBMCs or human monocytic cell line THP-1) revealed that lower concentrations of Cd showed stimulating effects on production of cytokines such as IL-1*β*, IL-6, and TNF-*α* [29, 30]. However, in the present study using activated murine RAW 264.7 macrophages as a highly standardized* in vitro* model, exposure to 10 *μ*M Cd did induce the secretion of IL-1*β*, too, whereas the production of IL-6 and IL-10 was significantly reduced. Lower expression of IL-10 might be explained by decreased IL-6 production [34]. Interestingly, production of TNF-*α* was not effected by Cd in our* in vitro* model. These results indicate that Cd effects on cytokine production depend on the cell type and also on the activation state of immune cells. In terms of TNF-*α*, the strong upregulation of this cytokine in activated RAW 264.7 macrophages might have overlain a possible TNF-*α*-inducing effect of Cd observed in nonactivated RAW 264.7 cells [31].

Additionally to IL-1*β*, also the secretion of chemokine CXCL1 (former designation in mouse: KC) was upregulated by exposure to 10 *μ*M but not to 0.1 *μ*M Cd. This upregulation was also reported by other groups for the homologous human chemokine IL-8 in human monocytic cell line THP-1 and in bronchial epithelial cells after exposition with Cd [30, 35]. Reduced IL-6 production is discussed as a possible explanation for upregulated secretion of CXCL1. Fielding et al. and Hurst et al. showed that IL-6 regulates CXCL1 expression via STAT3 resulting in recruitment of neutrophils [36, 37].

A decrease in NO production by 10 *μ*M Cd was also shown by other groups in LPS-stimulated RAW 264.7 macrophages and in murine splenic macrophages stimulated with TNF-*α*/IFN-*γ* [31, 38]. The reduced NO synthesis caused by 10 *μ*M Cd might be a significant reason for increased cell viability in activated macrophages, since it was previously shown that high cellular concentrations of NO may induce apoptosis in peritoneal macrophages [39]. But in conclusion, this result points out that macrophages exposed to such Cd concentrations might show impaired killing of living bacteria.

## 5. Conclusion

The data presented in this work deliver clear evidence for the capacity of Cd to modulate important cellular functions of activated macrophages as key players in innate immunity to infection in a strongly limited subtoxic dose range of this toxic heavy metal. In particular, the results found in our hk* S*.E.-stimulated RAW 264.7* in vitro* model indicate that Cd-mediated immunomodulation increases cell survival and inhibits the anti-inflammatory cytokines IL-6 and IL-10, while the secretion of proinflammatory cytokine IL-1*β* and the neutrophil-recruiting chemokine CXCL1 are induced by Cd. Although these findings alone are not sufficient to explain the higher susceptibility to* Salmonella* infection under Cd exposure as previously observed in a mouse model in our group but may contribute to explain the potential of long-term Cd exposure to elevate the risk of chronic inflammation following bacterial infection as previously concluded from epidemiologic studies.

## Figures and Tables

**Table 1:** Cadmium atoms per cell in RAW 264.7 macrophages. Cells were incubated for 2 h, 4 h or 20 h with 0.01 *µ*M, 0.1 *µ*M or 10 *µ*M CdCl_2_, or without CdCl_2_ for control, each in the presence or absence of hk *S*.E. Afterwards cells were harvested, washed, mineralized and the number of cadmium atoms per cell was determined by ICP-MS. Shown are the data derived from 2–6 independent biological replicates with standard deviation. (n.d. not determined)

	0 *µ*M CdCl_2_		0.01 *µ*M CdCl_2_		0.1 *µ*M CdCl_2_		10 *µ*M CdCl_2_	
	−hk *S*.E.	+hk *S*.E.	−hk *S*.E.	+hk *S*.E.	−hk *S*.E.	+hk *S*.E.	−hk *S*.E.	+hk *S*.E.
2 h	**2.63 ×**10^4^ ± 2 × 10^4^	**2.19 ×**10^4^ ± 1 × 10^4^	**1.13 ×**10^5^ ± 3 × 10^4^	**2.11 ×**10^5^ ± 1 × 10^5^	**1.04 ×**10^6^ ± 7 × 10^4^	**9.30 ×**10^5^ ± 3 × 10^4^	**1.65 ×**10^7^ ± 7 × 10^5^	**1.52 ×**10^7^ ± 2 × 10^6^

4 h	**1.35 ×**10^4^ ± 6 × 10^3^	**1.97 ×**10^4^ ± 1 × 10^4^	**2.20 ×**10^5^ ± 7 × 10^4^	**2.89 ×**10^5^ ± 1 × 10^5^	**2.25 ×**10^6^ ± 1 × 10^6^	**3.03 ×**10^6^ ± 2 × 10^6^	n.d.	n.d.

20 h	**4.02 ×**10^3^ ± 9 × 10^3^	**3.21 ×**10^4^ ± 4 × 10^4^	n.d.	n.d.	n.d.	n.d.	**1.81 ×**10^8^ ± 2 × 10^7^	**2.01 ×**10^8^ ± 1 × 10^7^

## Abbreviations

FBS: Fetal bovine serum
CI: Cell index
HEPES: 4-(2-Hydroxyethyl) piperazine-1-ethanesulfonic acid
hk* S*.E.: Heat-killed salmonellae
NO: Nitric oxide
ICP-MS: Inductively coupled plasma-mass spectrometry
ELISA: Enzyme-linked Immunosorbent Assay
PRR: Pattern-recognition receptor.
